# Basil Polysaccharide Reverses Development of Experimental Model of Sepsis-Induced Secondary Staphylococcus aureus Pneumonia

**DOI:** 10.1155/2021/5596339

**Published:** 2021-05-18

**Authors:** Xi Chen, Yue He, Qiang Wei, Chuanjiang Wang

**Affiliations:** ^1^Department of Laboratory Medicine, The First Affiliated Hospital of Chongqing Medical University, Chongqing, China; ^2^Department of Urology, North Kuanren General Hospital, Chongqing, China; ^3^Department of Critical Care Medicine, The First Affiliated Hospital of Chongqing Medical University, Chongqing, China

## Abstract

**Background:**

Basil polysaccharide (BPS) represents a main active ingredient extracted from basil (Ocimum basilicum L.), which can regulate secondary bacterial pneumonia development in the process of sepsis-mediated immunosuppression.

**Methods:**

In this study, a dual model of sepsis-induced secondary pneumonia with cecal ligation and puncture and intratracheal instillation of *Staphylococcus aureus* or *Pseudomonas aeruginosa* was constructed.

**Results:**

The results indicated that BPS-treated mice undergoing CLP showed resistance to secondary *S. aureus* pneumonia. Compared with the IgG-treated group, BPS-treated mice exhibited better survival rate along with a higher bacterial clearance rate. Additionally, BPS treatment attenuated cell apoptosis, enhanced lymphocyte and macrophage recruitment to the lung, promoted pulmonary cytokine production, and significantly enhanced CC receptor ligand 4 (CCL4). Notably, recombinant CCL4 protein could enhance the protective effect on *S. aureus*-induced secondary pulmonary infection of septic mice, which indicated that BPS-induced CCL4 partially mediated resistance to secondary bacterial pneumonia. In addition, BPS priming markedly promoted the phagocytosis of alveolar macrophages while killing *S. aureus in vitro*, which was related to the enhanced p38MAPK signal transduction pathway activation. Moreover, BPS also played a protective role in sepsis-induced secondary *S. aureus* pneumonia by inducing Treg cell differentiation.

**Conclusions:**

Collectively, these results shed novel lights on the BPS treatment mechanism in sepsis-induced secondary *S. aureus* pneumonia in mice.

## 1. Introduction

Sepsis is a complex immunopathological syndrome characterized by life-threatening organ dysfunction caused by a deregulated host response to systemic infection [1]. It is attributed to a persistent and complicated interaction of the proinflammatory process with the anti-inflammatory one in the body, leading to high inflammatory response and subsequent immune dysfunction [2, 3]. Globally, sepsis continues to be a major reason for deaths at intensive care unit (ICU) [4]. Recently, a global study reported approximately 49 million diagnosed patients along with 11 million deaths due to sepsis in the world in 2017, which accounted for around 20% total death cases globally. Furthermore, a study reported that the pooled incidence of hospital-treated sepsis patients was 189/100,000 person-years, whereas the estimated mortality rate was 26.7%. The study also reported that the prevalence of ICU-treated sepsis was 58/100,000 person-years, including 41.9% dying before hospital discharge. Notably, the incidence of hospital-treated sepsis considerably increased after 2008 [5]. Great inflammatory response is previously reported to induce sepsis-related deaths early, whereas compensatory anti-inflammatory response is suggested to cause deaths following organ failure via the dominant congenital immunity, affecting endothelial function, blood flow, and parenchymal cell metabolism [6]. However, recent studies have revealed that the persistent counterregulatory anti-inflammatory and proinflammatory state triggered by the imbalanced innate along with restrained adaptive immune responses leads to prolonged organ damage and dysfunction, leading to patient death [7]. Primary infections in patients with severe sepsis may not be the leading cause of death; however, persistent inflammation and immunosuppression represent the predominant cause of secondary infections and mortality [8]. In recent years, the increased prevalence of infection with antibiotic-resistant bacteria represents a significant challenge to the effective treatment of sepsis-induced secondary bacterial pneumonia in the hospital [9]. Pulmonary immunity exerts an important part in resisting the pulmonary respiratory pathogens, while different inflammatory mediators (such as chemokines, cytokines, or growth factors) modulate responses to various kinds of infection or injury [10]. Thus, further understanding pulmonary immunity together with the molecular and cellular immune responses upon microbial infection would significantly enhance our understanding of secondary lung infections' pathogenesis during the immunosuppressive phase of sepsis. Several studies have identified the association between suppression-mediated immunosuppression and secondary bacteria-induced pulmonary infection. Moreover, macrophage dysfunction [11], neutrophil paralysis [12], and lymphopenia [13] are related to secondary bacteria-induced pulmonary infection post-sepsis. Therefore, the immunosuppression induced by sepsis may markedly alter the modulation of pulmonary immunity in the host, resulting in the enhanced sensitivity among septic cases complicated by nosocomial pneumonia [14].

Basil or *Ocimum basilicum* L., belongs to the family Lamiaceae, is known as the “king of herbs” due to its extensive traditional use in medicine and for culinary and perfumery purposes worldwide. It is native to Southeast Asia, America, and parts of Africa and frequently planted within the gardens and pots across Southwest Asia, the USA, and Europe [15]. Basil has been shown to exhibit potential pharmacological effects, including anticancer, antistress, antidiabetic, antipyretic, antioxidant, immunomodulatory, hypolipidemic antiatherosclerotic effect, and antibacterial activities [16–19]. Among the essential active compounds of basil, basil polysaccharide has been shown to exhibit a variety of pharmacological activities [20]. Studies have demonstrated that basil polysaccharide (BPS) is adopted to be the immunopotentiator for stimulating macrophages, protecting immune organs, while building the complement system for exerting immune enhancement effects. Moreover, basil polysaccharide exhibits good antibacterial activity [21]. BPS can also inhibit various bacteria infected in clinic [22, 23]. Currently, BPS has been extensively utilized to lower blood lipids, prevent atherosclerosis, and treat cancer and diabetes [24, 25]. However, there is a paucity of literature on the effects of basil polysaccharide on sepsis-induced secondary bacterial infection in the lungs.

Hospital-acquired secondary pneumonia, a frequent nosocomial bacterial infection, accounts for a major reason leading to deaths among severe sepsis cases [26]. Organisms causing hospital-acquired secondary pneumonia leading to severe sepsis are dominated by *Staphylococcus aureus* (20.5%), followed by *Pseudomonas* species (19.9%), fungi (19%), and Enterobacter (mostly *Escherichia coli*, 16.0%) [27]. Herein, a dual model of sepsis-induced secondary pneumonia with cecal ligation and puncture (CLP) along with intranasal instillation of *Pseudomonas aeruginosa* or *Staphylococcus aureus* was established to elucidate the effects of basil polysaccharide in sepsis-induced secondary lung bacterial infection.

## 2. Materials and Methods

### 2.1. Animals

The 8-12-week-old C57BL/6 male mice (weight, 20-24 g) were provided by Laboratory Animal Center of Chongqing Medical University (Chongqing, China). The license number is SYXK (Chongqing, China) 2018-0003. Thereafter, all animals were raised in the specific pathogen-free (SPF) environment under 24°C, 50%-60% relative humidity (RH), and 12 h/12 h light/dark cycle conditions. Each mouse was allowed to drink water and eat standard food. Each animal was healthy and infection-free throughout the experiment.

All mice were treated following the Guidelines for the Care and Use of Laboratory Animals in China. The Institutional Animal Care and Use Committee of Chongqing Medical University approved our study protocol.

### 2.2. “Double-Hit” Mice Model

CLP and intratracheal injection of *S. aureus* or *P. aeruginosa* were carried out as the first and second hits, respectively. Briefly, each mouse was given intraperitoneal injection of ketamine (1 mg/ml) and 100 *μ*l xylazine (20 mg/ml) contained within PBS for anesthesia, followed by cecal ligation and puncture using the 26G needle (nonsevere CLP, resulting in the mortality rate of 5%–10% in WT mice). Later, we put back the cecum into peritoneal cavity, followed by incision closure using the surgical staples. All mice were given subcutaneous administration of 0.9% sterile normal saline at the dose of 5 ml/100 g body weight (BW) preheated at 37°C for replacing the 3^rd^ space loss; thereafter, the warm pad was prepared for resuscitation [28].

At 3 days after CLP, the xylazine/ketamine mixture was administered into the surviving mice for anesthesia. Then, each mouse was placed in the “head-up” position, and the trachea was exposed, followed by intratracheal injection (i.t.) with *P. aeruginosa* (5 × 10^7^ colony-forming units (CFUs) within 50 *μ*l PBS) or *S. aureus* (5 × 10^7^ CFUs within 50 *μ*l PBS) [29].

### 2.3. *In Vivo* Administration of Basil Polysaccharides

For *in vivo* basil polysaccharide treatment, each mouse was administered i.p. with 75 mg/kg of basil polysaccharides [30] (Shanxi kingreg Biotech. Ltd., China) or IgG 2 h after the second hit. With regard to CCL4 exposure *in vivo*, all animals were given 500 ng IgG or recombinant mouse CCL4 (R&D Systems, USA) i.p. at the time of the second hit of *S. aureus*.

### 2.4. Lung Tissue and Bronchoalveolar Lavage Fluid Collection

At 24 h following S. aureus or P. aeruginosa i.t., the animals were killed under anesthesia. Lungs were extracted, and tissues were harvested, followed by the immediate collection of bronchoalveolar lavage fluid (BALF). After chest clapping, right bronchial bundling and left lung lavage were carried out. In addition, after resecting the right lung, we obtained the right upper lobe to count the bacterial numbers, whereas the rest right lung tissues were preserved under −70°C at once for further analysis.

### 2.5. Determination of Lung and Plasma Bacterial Burdens

Plasma samples were obtained at specific time periods. Meanwhile, we also resected the right upper lung lobe under aseptic condition, followed by homogenization within 1 ml sterile saline using the tissue homogenizer by the use of a vented hood. Later, we diluted plasma and lung homogenate at serial concentrations. For every dilution, 10 ml sample was added on the predried tryptic soy-base blood agar plates, followed by overnight incubation under 37°C. Afterwards, CFUs were counted and expressed as total CFU per lung or per milliliter of plasma.

### 2.6. Measurement of Inflammatory Mediators

Blood samples were collected in heparinized tubes *via* the ophthalmic vein. Inflammatory mediators, such as CCL4, IL-10, CXCL-1, TNF-*α*, IL-1*β*, IL-6, and IL-17A, were assessed by the Mice Cytokine Magnetic Bead Panel Kit (eBioscience, USA) following the manufacturer's protocol.

### 2.7. Determination of Chemokine CCL4 Produced by Neutrophils

The neutrophils were sorted from the bronchoalveolar lavage using magnetic separation (Miltenyi Biotec) and were suspended in 10% FBS (Sigma, USA) and RPMI1640 (Sigma, USA) and then inoculated on the culture plate. To determine whether basil polysaccharides promote the secretion of CCL4 by neutrophils, we supplemented basil polysaccharide [31] (100 *μ*g/ml, kingreg Biotech, China) or PBS to the culture. After incubation for 48 h, the chemokine CCL4 in the supernatant was quantified by ELISA using kits (R&D, USA) following specific protocols. The absorbance of each sample was read at 450 nm.

### 2.8. Lung Injury Index Assessment

Lung injury index assessment is as follows: (1) Morphological evaluation: as for the right upper lung lobe, it was subjected to 10% formalin fixation, paraffin embedding, and sectioning into 4 *μ*m sections. Then, the sections were deparaffinized, dehydrated, and stained by hematoxylin and eosin (H&E) to carry out histological examinations. Mikawa's method was adopted to estimate lung injury score by adopting the 4 indicators below: (1) alveolar hyperemia, (2) hemorrhage, (3) neutrophil or interstitial aggregation or infiltration, (4) hyaline membrane formation or alveolar septal thickening, where 0-4 marks indicated no/very mild, mild, moderate, severe, and very severe damage, respectively. All scores were added up as the final score, and the ARDS pathological score was indicative of increases in lesion number. Lung injury was rated according to the 0-4 scale based on lesion severity of every indicator, where 0-4 points indicated normal results, mild (<25%), moderate (25-50%), severe (50-75%), and very severe (>75%) lung involvements, separately. A greater score was indicative of the more severe lesion. The light microscope (Olympus, Japan) was utilized to evaluate the abnormal histological results. (2) Albumin assessment: albumin for lung permeability assessment was performed using a Albumin Quantification Kit (Bethyl Laboratories, Montgomery, TX) following specific protocols. (3) Myeloperoxidase (MPO) measurement: the MPO activity in the tissue was measured to quantify lung neutrophil infiltration. In brief, we homogenized lung tissues with the 20 mmol/l PBS (pH 7.4), followed by 10 min of centrifugation at 4°C and 10,000 g. Later, pellets were resuspended with 50 mmol/l PBS (pH 6.0) contained within 0.5% hexadecyltrimethylammonium bromide (Sigma), and then, the homogenate was treated with 4 freeze-thawing cycles, followed by 40 s of sonication for disruption. Afterwards, the samples were subjected to 5 min of centrifugation for 40 s at 10,000 g and 40,000 ion. The sample was assayed for the myeloperoxidase activity according to previous description, with tetramethylbenzidine (Sigma) being the substrate. Later, we detected the absorbance (OD) values at 460 nm and adjusted them based on tissue weights (fold change (FC) relative to control). (4) Wet/dry weight: after dissecting left lung, we weighed the wet weight. The lung was incubated, then dried in an oven at 60°C for 3–4 days and reweighed as dry weight. Then, the wet weight was divided by dry weight to calculate the wet-to-dry (W/D) weight ratio [32]

### 2.9. TUNEL Assay

The In situ Cell Apoptosis Detection Kit I, POD (Roche, Switzerland) was utilized to measure cell apoptosis rate by TUNEL assay following specific instructions. In brief, after xylene deparaffinage, the 4 *μ*m sections were subjected to gradient ethanol rehydration. Thereafter, 3% hydrogen peroxide (H_2_O_2_) was used to block endogenous peroxidase activity for a period of 10 min; afterwards, 10–20 *μ*g/ml proteinase K solution was utilized to digest sections under 37°C for 15 min. After PBS washing, terminal deoxynucleotidyl transferase diluted at 1 : 20 supplemented within the reaction buffer (digoxigenin-labeled nucleotides) was used to react with sections for 2 h under 37°C. Thereafter, the stop/wash buffer was used to rinse slides for 2 min thrice. Subsequently, antidigoxin antibody previously diluted at 1 : 100 was used to incubate sections under 37°C for 30 min, and later, ABC was employed to further incubate sections for 30 min under 37°C. Apoptosis was measured through incubating sections using 3,3′-diaminobenzidine chromogen for about 20 min, followed by hematoxylin counterstaining. Later, 5 fields of view (FOVs) were selected randomly from every section (×400 magnification). Then, TUNEL-positive cell proportion per field was recorded at ×400 magnification in 5 random fields.

### 2.10. Western Blot Analysis

The protein extraction kit (Beyotime, China) was utilized to extract total macrophage proteins in accordance with specific protocols. Bicinchoninic acid (BCA) protein assay kit (Pierce, USA) was employed for detecting protein contents. Thereafter, proteins were separated through 10%SDS-PAGE, followed by transfer to the nitrocellulose membranes. After transfer, the membranes were incubated in blocking buffer containing 5% (*w*/*v*) skimmed milk supplemented within the Tris-buffered saline that contained 0.05% Tween-20, followed by overnight incubation with primary antibody under 4° C and then secondary antibody incubation. At last, the ECL detection system was used to visualize protein blots.

### 2.11. Flow Cytometry

After PBS washing, cells were prepared into pellets and analyzed by the flow cytometer. The following monoclonal antibodies including CD4, CD25, Foxp3, CD11b, Ly6G, F4/80. To stain CD4, CD25, Foxp3, CD11b, Ly6G, F4/80, the Fixation/Permeabilization kit (eBioscience, USA), anti-CD4-FITC, anti-CD11b-APC, anti-Ly6G-FITC, anti-F4/80-FITC, anti-CD25-PE, and anti-Foxp3-APC (eBioscience, USA) were utilized following specific protocols. The FACScan flow cytometer (Becton Dickinson) was used to collect cells (10^5^), whereas FlowJo software 7.6 was adopted for analysis.

### 2.12. Cell Purification and Culture

We adopted the Lymphocyte Separation Medium (GE healthcare, USA) to isolate splenic peripheral blood mononuclear cells (PBMCs) from mice. Thereafter, magnetic activated cell sorting (Miltenyi Biotec) was carried out to isolate naïve CD4+ T cells from PBMCs using the Naïve CD4+ T Cell Isolation Kit II (StemCell, Canada) following specific protocols. Then, flow cytometric analysis was performed to measure the naïve CD4+ T cell purity (> 90%). Then, we cultivated cells within the RPMI 1640 complete medium (Gibco, Grand Island, NY, USA) that contained 10% fetal bovine serum (FBS) and incubated them under 37°C and 5% CO_2_ conditions.

### 2.13. Treg Cell Subset Generation

In this study, we produced Treg cell subsets through exposing to 50 mM *β*-mercaptoethanol, 2 mM L-glutamine, 2 *μ*g/ml anti-CD28, 5 *μ*g/ml anti-CD3, 2.5 ng/ml TGF-*β*, and 50 U/ml IL-2 for a period of 3 days. To determine whether basil polysaccharide was involved in the induction process, we supplemented basil polysaccharide (100 *μ*g/ml, kingreg Biotech, China) to the culture. Flow cytometric analysis was performed to assess intracellular staining and surface marker expression.

### 2.14. Macrophage Phagocytosis Assays

BALFs were incubated using 0.5 mg/ml FITC (Sigma) under 37°C for 20 min, so that macrophages adhered to the plastic, FITC-labeled S. aureus for separation. Thereafter, FITC-labeled bacteria (MOI, 100) were used to incubate the separated macrophages under 37°C for 30 min. Then, cells were washed, and nuclei were subjected to DAPI (Invitrogen) staining and visualized under the confocal laser scanning microscope (LSM 510, Zeiss). One independent reviewer was responsible for quantifying engulfed bacterial proportion of the 300 cells counted/well. For certain experiments, 100 *μ*g/ml BPS (kingreg Biotech, China) was used to pretreat bronchoalveolar macrophages before infection with FITC-labeled *S. aureus*.

### 2.15. Macrophage Killing Assays

Alive *S. aureus* (with the multiplicity of infection (MOI) of 10) was used to infect 1 × 10^5^ bronchoalveolar macrophages for 1 h under 37°C. Later, buffer that contained 100 *μ*g/ml tobramycin was adopted to wash cells for removing extracellular bacteria, whereas lysis buffer (Promega) was used for lysis. Lysate culture was utilized to quantify alive intracellular bacteria so as to assess bacterial uptake as well as intracellular killing (*t* = 0 and 2, respectively). Killing was determined by colony proportion occurring at *t* = 2 h in comparison with that at *t* = 0 h,100 − [CFU number at *t* = 2 h/CFU number *t* = 0 h]. In certain experiment, 100 *μ*g/ml BPS (kingreg Biotech, China) was used to pretreat bronchoalveolar macrophages prior to alive S. aureus infection.

### 2.16. Statistical Analysis

SPSS19.0 (IBM, Armonk, New York, USA) was employed for statistical analysis. Values were presented in the manner of median (interquartile ranges) or mean ± SD. Differences of two groups were evaluated by Mann–Whitney *U* tests, whereas those among several groups were evaluated by one-way ANOVA. Log-rank (Mantel-Cox) test was used to analyze survival curves. *p* < 0.05 indicated statistical significance.

## 3. Results

### 3.1. Basil Polysaccharide Can Significantly Improve the Prognosis of the Sepsis-Induced Secondary *S. aureus* Pneumonia Mice Model, but Not in Secondary *P. aeruginosa* Pneumonia

**Figure 1:**
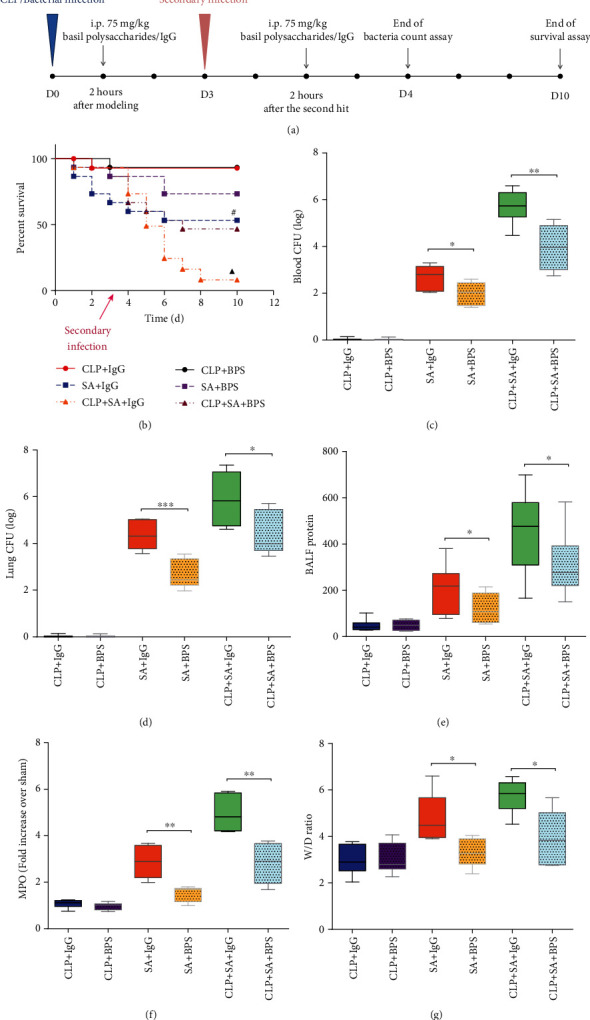
(a) Experimental procedure. We randomized mice into 6 groups, including 4 receiving CLP at D0 and 2 receiving sham operation. At 3 days later (D3) or at D0 in the sham operation group, mice were given intratracheal injection with *P. aeruginosa* (PA, 5 × 10^7^ CFU) or *S. aureus* (SA, 5 × 10^7^ CFU). Two hours after the bacterial hit or the second bacterial hit, basil polysaccharide or IgG was injected intraperitoneally as an intervention. We collected lung tissues, blood, and BALF at 24 h after a bacterial infection or secondary bacterial infection for analysis. In the 10-day experimental period, we recorded the mortality rates of all groups to analyze the survival. (b) The mortality rates were monitored for 10 days after the challenge with *S. aureus* (*n* = 15 mice/group). (c, d) Lung or blood bacterial CFU in each group after administration with *S. aureus* (*n* = 5 mice/group). (e–g) Lung injury assessment indicators such as protein in BALF, myeloperoxidase, and wet/dry weight ratio in the lung were measured after challenge with *S. aureus* (*n* = 5 mice/group). Log-rank (Mantel-Cox) test was performed to analyze survival curves. Values were presented in the manner of mean ± SD, while one-way ANOVA as well as LSD multiple comparisons test was adopted for data analysis. ^#^*p* < 0.05, compared with *S. aureus* infection treated with basil polysaccharide. ^▲^*p* < 0.05, compared with CLP-surgery mice upon secondary *S. aureus* infection treated with basil polysaccharide. ^∗^*p* < 0.05, ^∗∗^*p* < 0.01, and ^∗∗∗^*p* < 0.001, upon one-way ANOVA as well as LSD multiple comparisons. Compared with *S. aureus* infection treated with basil polysaccharide group or CLP-surgery mice upon secondary *S. aureus* infection treated with the basil polysaccharide group.

For investigating the possible effect of BPS on the sepsis-mediated secondary bacterial pulmonary infection, we treated C57BL/6 mice with CLP, followed by intratracheal injection with bacteria (*S. aureus* or *P. aeruginosa*) and BPS or IgG treatment. The entire experimental design and procedures were presented in Figure 1(a). As shown in Figures 1(b)–1(g), in the CLP-induced nonsevere sepsis model, survival rate between BPS-exposed and IgG control groups showed no significant difference, and their survival rate was about 90%. Therefore, there was no significant difference in mouse lung injury indicators such as protein in BALF, MPO, and W/D ratio. However, in the bacterial pneumonia model, mice's mortality began to increase, and the mortality was the highest in the CLP-induced secondary bacterial pneumonia mouse model. Next, we found that basil polysaccharide administration can improve the survival rate of *S. aureus* pneumonia or CLP-induced secondary *S. aureus* pneumonia mouse model. Moreover, it can also reduce the bacterial load in mice's blood and lungs and improve lung injury indicators. However, these results were not observed in *P. aeruginosa* pneumonia or CLP-induced secondary *P. aeruginosa* pneumonia mice model (supplementary data (available here)).

### 3.2. CLP Resulted in Impaired Host Pulmonary Immunity in the Mice

**Figure 2:**
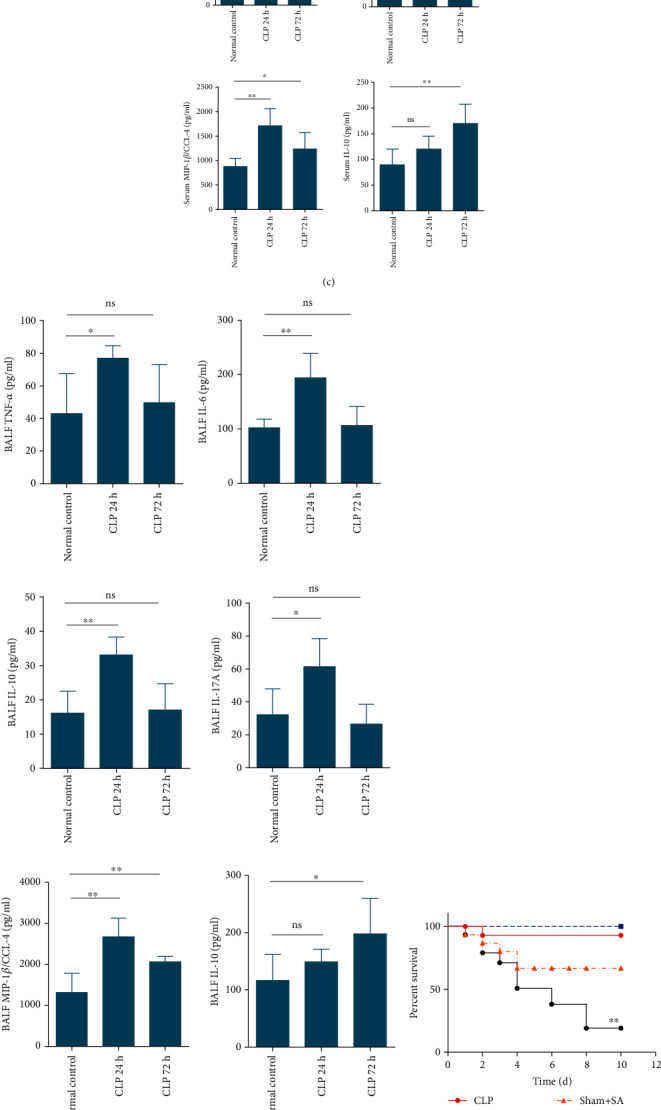
CLP led to damaged pulmonary immune responses in the host. Mice receiving CLP or sham operation. (a, b) Histological scores for CLP-induced nonsevere sepsis model (*n* = 5 mice/group). (c, d) At 24 h and 72 h after CLP, we detected contents of cytokines in serum and BALF. Mice Cytokine Magnetic Bead Panel Kit (*n* = 5 for every group) was performed to analyze the obtained specimens. (e) 72 h after CLP, mice were given intratracheal injection with *S. aureus* (5 × 10^7^ CFU). Following challenge (*n* = 15 for every group), we observed mortality rates over the 10-day period. Log-rank (Mantel-Cox) test was performed to analyze survival curves. (f) At 24 h following secondary infection with *S. aureus*, we detected contents of chemokines and cytokines in serum and BALF. Mice Cytokine Magnetic Bead Panel Kit (*n* = 5 for every group) was performed to analyze the obtained specimens. Values were presented in the manner of mean ± SD, whereas nonparametric Mann–Whitney *U* test was adopted for data analysis. ^∗^*p* < 0.05, ^∗∗^*p* < 0.01, compared with normal control or sham-surgery mice upon secondary S. aureus infection.

For confirming the effect of CLP on attenuating pulmonary response based on the microbial sepsis model, firstly, we detected the mice of immune status after CLP. The results showed that 24 hours after CLP, lung injury and inflammatory mediators including MIP-1*β*/CCL4, IL-10, TNF-*α*, IL-1*β*, IL-6, and IL-17A in serum or BALF were increased significantly. However, 72 hours after CLP, the lung injury was gradually recovered. Proinflammatory cytokines including MIP-1*β*/CCL4, TNF-*α*, IL-1*β*, IL-6, and IL-17A in serum or BALF were decreased, while the anti-inflammatory cytokine IL-10 continued to increase (Figures 2(a)–2(d)). Next, we treated WT C57BL/6 mice with sham operation or CLP, followed by intratracheal infection by *S. aureus* at 72 h after CLP. All mice undergoing sham operation survived, whereas over 90% mice receiving CLP with the 26G needle survived. Nonetheless, after *S. aureus* intrapulmonary administration at 5 × 10^7^ CFU, 67% animals in the sham operation group survived. On the contrary, most animals exposed to sublethal CLP died upon subsequent intratracheal injection of *S. aureus* (Figure 2(e)). In addition, animals subjected to CLP that developed secondary *S. aureus* pneumonia showed markedly reduced BALF or serum inflammatory mediator production, such as IL-1*β*, IL-6, IL-17A, TNF-*α*, and MIP-1*β*/CCL4, whereas upregulated anti-inflammatory mediator (IL-10) production relative to the sham operation group, and pneumonia occurred at 24 h following infection (Figure 2(f)). Collectively, the above results conformed to previous results suggesting that CLP led to compromised pulmonary immune response upon secondary *S. aureus* infection.

### 3.3. Basil Polysaccharide Protected Mice from Lethality, Ablated Lung Pathology, and Regulated Inflammatory Responses in Sepsis-Induced Secondary *S. aureus* Pneumonia Mice Model

**Figure 3:**
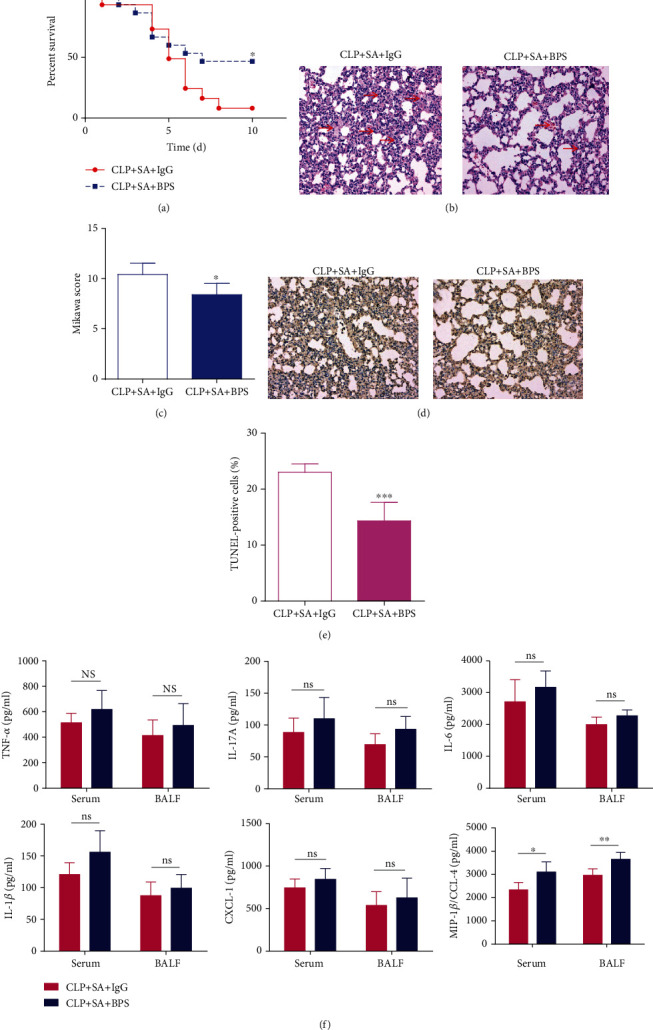
Postseptic basil polysaccharide is resistant to *S. aureus* pneumonia. (a) Survival of mice treated with basil polysaccharide upon *S. aureus* infection during sepsis (*n* = 15 mice/group). (b) Typical HE staining for lung tissue samples at 24 h postinfection with *S. aureus* during sepsis and following treatment with IgG or basil polysaccharide group. (c) Histological scores for secondary pulmonary infection with *S. aureus* within septic mice, as well as following treatment with IgG or basil polysaccharide (*n* = 5 mice/group). (d, e) TUNEL assay was performed to determine cell apoptosis, where the nuclei of TUNEL-positive cells were dark-brown. (f) BALF and serum cytokine or chemokine contents were detected at 24 h after treatment with IgG or basil polysaccharide during sepsis-induced secondary *S. aureus* pneumonia in mice. Specimens were collected for analysis by Mice Cytokine Magnetic Bead Panel Kit (*n* = 5 mice/group). Survival curves were analyzed using the log-rank (Mantel-Cox) test. Data were expressed as mean ± SD, whereas nonparametric Mann–Whitney *U* test was applied for data analysis. ^∗^*p* < 0.05, ^∗∗^*p* < 0.01, and ^∗∗∗^*p* < 0.001, relative to secondary pulmonary infection with *S. aureus* of septic mice in the IgG group.

To assess the involvement of basil polysaccharide in host defense against *S. aureus* in septic mice, IgG or basil polysaccharide was administered to intervene in mice. The results revealed that the basil polysaccharide-treated mice group receiving CLP had remarkably elevated survival rate after secondary *S. aureus* infection, relative to the IgG group (Figure 3(a)). From the lung histopathological examination, in mice treated with basil polysaccharide, the lung injury scores were significantly reduced, indicated by improved hemorrhage, edema, and inflammatory cell infiltration in the CLP-induced secondary *S.aureus* pneumonia mouse model (Figures 3(b) and 3(c)). Additionally, pulmonary TUNEL-positive cell proportion declined following BPS exposure (Figures 3(d) and 3(e)). As presented in Figure 3(f), although there was no statistical significance, the basil polysaccharide-treated group exhibited comparatively increased chemokine or cytokine production (such as CXCL1, TNF-*α*, IL-6, IL-1*β*, and IL-17A) within alveolar lavage fluid and serum, compared with the IgG group, with statistically significant differences in CCL4 levels. Together, these findings indicated that the therapeutic effect of basil polysaccharide may be related to the recruitment of chemokines CCL4 in the lungs.

### 3.4. Effects of Basil Polysaccharide on Leukocyte Recruitment in Sepsis-Induced Secondary *S. aureus* Pneumonia Mice

**Figure 4:**
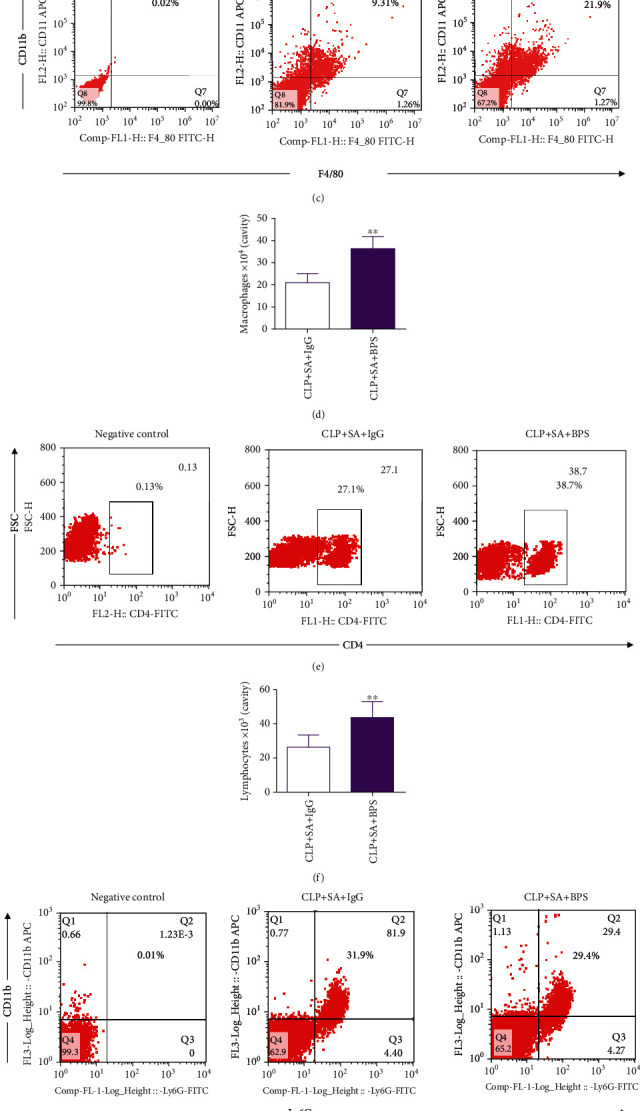
(a, b) Gating strategy to analyze total cells within BALF. (c, d) Gating of CD11b+F4/80+ cells was conducted to determine overall macrophage count within BALF. (e, f) Gating of CD4+ cells was conducted to determine overall T lymphocyte count within BALF. (g, h) Gating of CD11b+Ly6G+ cells was conducted to determine overall neutrophil count within BALF. Kaplan–Meier analysis and log-rank tests were conducted to compare two groups. ^∗^*p* < 0.05, ^∗∗^*p* < 0.01, compared with secondary *S. aureus* pneumonia in septic mice treated with the isotypical IgG control.

For identifying the possible mechanisms of BPS in changing the antibacterial defense in the host, this study measured the leukocyte influx into primary infection site following sepsis-mediated secondary *S. aureus* pulmonary infection. The overall cell number within mouse alveolar lavage fluid (BALF) increased significantly after treatment with basil polysaccharide (Figures 4(a) and 4(b)). Notably, treatment with basil polysaccharide significantly enhanced lymphocyte and macrophage counts within BALF relative to the IgG treatment group (Figures 4(c)–4(f)). On the contrary, differences in overall neutrophil count were not significant (Figures 4(g) and 4(h)). These results collectively suggest that the protection of basil polysaccharide during infection is still crucial to recruit lymphocytes and macrophages in this model.

### 3.5. Basil Polysaccharides Improve the Survival Rate of Sepsis-Induced Secondary *S. aureus* Pneumonia Mice by Promoting CCL4 Secretion from Neutrophils

**Figure 5:**
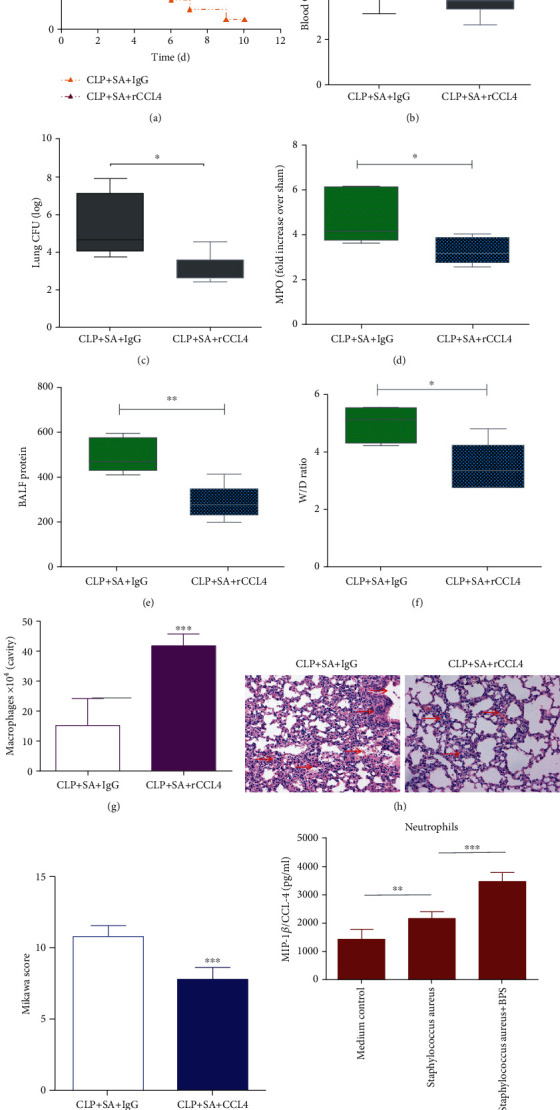
Effect of recombinant protein CCL4 (CC receptor ligand 4) on resistance in septic mice with *S. aureus* pneumonia. Recombinant protein CCL4 was administered (500 ng) 2 hours after *S. aureus* inoculation in septic mice. The control group was given equivalent IgG control. (a) Survival of septic mice with secondary *S. aureus* infection (*n* = 15 mice/group) following recombinant protein CCL4 administration. (b, c) Blood and lung CFU of septic mice with secondary *S. aureus* infection (*n* = 5 mice/group) following recombinant protein CCL4 administration. (d–f) Lung damage assessment indicators such as protein in BALF, myeloperoxidase (MPO), and wet/dry weight ratio in septic mice with secondary *S. aureus* infection (*n* = 5 mice/group) following recombinant protein CCL4 administration. (g) The total number of macrophages in BALF in septic mice with secondary *S. aureus* infection (*n* = 5 mice/group) following recombinant protein CCL4 administration. (h, i) Histological scores for secondary pulmonary infection with *S. aureus* of septic mice (*n* = 5 for every group). Log-rank (Mantel-Cox) test was performed to analyze survival curves. Values were presented in the manner of mean ± SD, whereas nonparametric Mann–Whitney *U* test was adopted for data analysis. ^#^*p* < 0.05, ^∗^*p* < 0.05, ^∗∗^*p* < 0.01, and ^∗∗∗^*p* < 0.001, relative to secondary pulmonary infection with *S. aureus* of septic mice receiving recombinant protein CCL4 treatment. (j) The concentration of CCL4 in the cell supernatant after *S. aureus* or basil polysaccharide stimulates neutrophils for 48 hours. ^∗∗^*p* < 0.01, ^∗∗∗^*p* < 0.001, upon one-way ANOVA and LSD multiple comparisons, relative to the *S. aureus* group.

Previous studies have found that the chemokine CCL4 exerts a vital part in the pathogenic mechanism of pulmonary diseases like bacterial pneumonia and respiratory defense [33]. Our study revealed that basil polysaccharide can significantly increase the level of CCL4 in the lungs of sepsis-induced secondary *S. aureu*s pneumonia mice (Figure 3(f)). This indicated that the therapeutic effect of basil polysaccharide may be related to the recruitment of chemokine CCL4 in the lungs. Therefore, we investigated the role of CCL4 in sepsis-induced secondary *S. aureus* pneumonia mouse model. First, we observed that in secondary *S. aureus* pneumonia induced by sepsis, recombinant CCL4 could improve lung pathology and lung injury, increase the clearance rate of bacteria from the lung and blood, reduce lung injury and mortality, and effectively promote macrophage recruitment in the lungs (Figures 5(a)–5(i)). Neutrophils are immune cells that can secrete a variety of chemokines, such as IL-1*β*, IL-8, interferon-*γ* inducible protein 10 (IP-10), and CCL4 [32]. Although we did not identify the ability of basil polysaccharide in promoting neutrophil recruitment in the lungs, *in vitro* experimental results revealed that basil polysaccharide could effectively promote the secretion of CCL4 by neutrophils. These findings highlighted the molecular immune mechanism of basil polysaccharide in regulating sepsis-induced secondary *S. aureus* pneumonia in mice (Figure 5(j)).

### 3.6. Basil Polysaccharide Induces Macrophage Phagocytosis and Killing *S. aureus* by p38 MAPK Signaling Pathway

**Figure 6:**
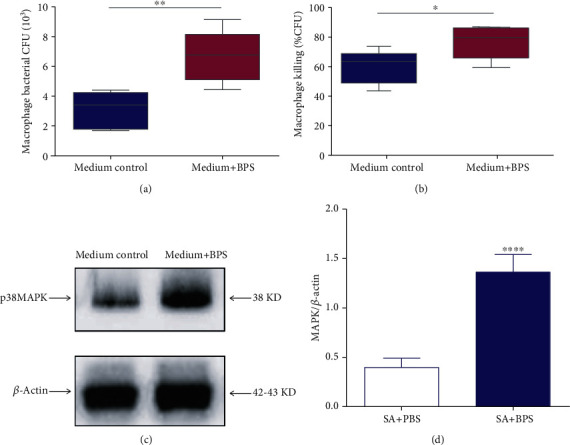
Effects of basil polysaccharide treatment on the ability of macrophages to eliminate and swallow bacteria. (a, b) 12 h BPS treatment was conducted on macrophages, followed by 30 min of S. aureus (MOI, 10) or FITC-labeled *S. aureus* infection under 37°C. We then determined the swallowed FITC-labeled *S. aureus* count and intracellular bacterial killing (*t* = 2 h) according to specific descriptions. (c, d) After 12 h of BPS treatment, Western blotting assay was conducted to determine p38MAPK signals within macrophages. ^∗^*p* < 0.05, ^∗∗^*p* < 0.01, and ^∗∗∗∗^*p* < 0.0001, upon one-way ANOVA as well as LSD multiple comparisons, in comparison with the BPS group.

To determine whether basil polysaccharide induced the inherent bacteria defense ability of phagocytes, this study examined bacterial absorption and macrophage clearance in the bronchoalveolar lavage fluid. Pretreatment with basil polysaccharide promoted phagocytosis and intracellular killing of *S. aureus* by macrophages (Figures 6(a) and 6(b)). Moreover, this study explored the possible mechanism by which BPS affected the S. aureus killing and phagocytosis abilities. The p38 MAPK signaling pathways exert vital parts in the regulation of bacterial clearance and macrophage phagocytosis [32, 34]. As a result, this study conducted Western blotting assay for analyzing the expression of proteins related to such signal transduction pathways. Following BPS treatment, the p38 MAPK signal expression increased significantly (Figures 6(c) and 6(d)).

### 3.7. Basil Polysaccharides Promote the Differentiation of Regulatory T Lymphocytes in Sepsis-Induced Secondary *S. aureus* Pneumonia Mice

**Figure 7:**
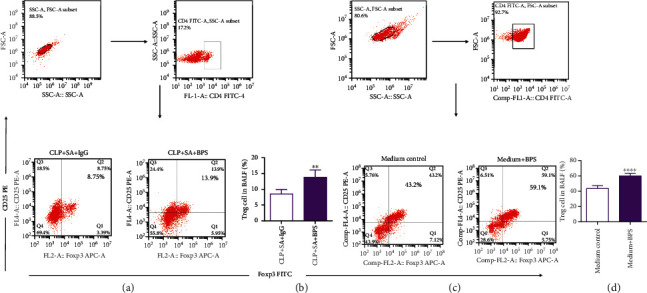
BPS promoted naive CD4 T lymphocytes to differentiate to Treg cells. (a, b) We separated T cells from BALF of mice. CD4+CD25+Foxp3 Treg cell proportion was then measured through flow cytometric analysis, where the findings indicated the means from 5 mice at each time point. (c, d) Treg proportion elevates relative to the nontreatment group following BPS challenge. ^∗∗^*p* < 0.01, ^∗∗∗∗^*p* < 0.0001, one-way ANOVA as well as LSD multiple comparisons test was conducted to compare two groups.

The previous results found that CD4^+^lymphocytes increased significantly in sepsis-induced secondary *S. aureus* pneumonia mice (Figures 4(e) and 4(f)). Next, we used flow cytometry to detect Treg lymphocytes in mouse BALF. The results revealed that after basil polysaccharide administration, the Treg cells in mice BALF increased significantly (Figures 7(a) and 7(b)). In order to further analyze the effect of basil polysaccharide on the differentiation of Treg lymphocytes, naïve CD4+ T lymphocytes were isolated from the mouse spleens and cultured *in vitro*. Afterwards, cells were intervened with BPS. At 3 days later, a trend of differentiation to Treg cells was observed among the naïve CD4+ T lymphocytes (Figures 7(c) and 7(d)). Taken together, these data demonstrated that basil polysaccharide could promote naïve CD4+ T lymphocytes to differentiate to Treg cells, thus exerting the immunomodulatory effect in sepsis-induced secondary *S. aureus* pneumonia mice.

## 4. Discussion

Following clinical cure, patients with microbiologic treatment failure experience significantly high rates of recurrent pneumonia and high susceptibility to sepsis-induced secondary lung infection [35]. These findings have been associated with the development of sepsis-induced immunosuppression [36]. Several new therapies have been reported to reduce sepsis-induced immunosuppression rates and limit the susceptibility to secondary pneumonia in recent years. However, these new approaches' efficacy remains poor and represents a significant challenge for clinicians [37]. Great attempts have been tried to avoid antimicrobial resistance spread; the development of resistant bacteria remains inevitable over time [38]. A possible method is related to the immunity-specific targeted treatment [39]. Basil, with diverse medicinal applications, has been incorporated into the Pharmacopeia (2015 edition). Basil polysaccharide is considered the most important active compound of basil, whereas mannose (Man), rhamnose (Rha), glucose (Glc), fructose (Fru), and Arabian sugar (Ara) represent its main components [40–42]. Studies have revealed that polysaccharides may be adopted to be immunopotentiators for stimulating macrophages, protecting immune organs, in the meantime of building the complement system, thus exerting the role of immunoenhancers [43, 44]. Not only that, basil polysaccharide also exhibits a wide range of antibacterial activities. They also exhibit inhibitory effects on a variety of common bacterial infections [45]. In this study, we observed that in experimental sepsis-induced secondary *S. aureus* pneumonia model, basil polysaccharides could improve lung pathology and lung injury, increase the clearance rate of bacteria from the lungs and blood, and effectively reduce mortality(Figure 1); however, no such effects were observed in experimental sepsis-induced secondary *P. aeruginosa* pneumonia model (Supplementary data (available here)). These findings indicate that basil polysaccharide could serve as a new type of adjuvant treatment to sepsis-induced secondary *S. aureus* pneumonia.

The out-of-balance between proinflammatory cytokine levels and anti-inflammatory cytokine levels is a characteristic of sepsis-mediated immunosuppression, and this makes the host susceptible to secondary pneumonia, especially nosocomial pneumonia [46, 47]. In general, as presented in Figures 2(c) and 2(d), at 72 hours after CLP, proinflammatory cytokines including MIP-1*β*/CCL4, TNF-*α*, IL-1*β*, IL-6, and IL-17A in serum or BALF were decreased and the anti-inflammatory cytokine IL-10 was significantly increased. Meanwhile as compared with the control group (sham+SA), the alveolar lavage fluid and serum samples of mice in the CLP+SA group revealed lower levels of proinflammatory cytokines or chemokines (including TNF-*α*, IL-1*β*, IL-17A, IL-6, and CCL-4) and higher levels of anti-inflammatory cytokines (IL-10), indicating that the sepsis-induced secondary S. aureus pneumonia mouse model presented an immunosuppressive state (Figure 2(f)).

Next, we intervened by administrating basil polysaccharide 2 hours after the second hit. As shown in Figure 3(f), although there is no statistical significance, the basil polysaccharide-treated group exhibited slightly increased chemokine and cytokine expressions (such as IL-1*β*, TNF-*α*, IL-6, IL-17A, and CXCL1) within the alveolar lavage fluid and serum samples, compared with the IgG group, with statistically significant differences in CCL4 levels. Together, these findings indicate that the therapeutic effect of basil polysaccharide may be related to the recruitment of chemokine CCL4 in the lungs. As for host defense, recruiting immune and inflammatory effector cells into tissue injury, neoplasia, and infection sites is still an important part. Such response can be partially modulated through the locally produced mediator network, such as lipids or chemotactic proteins [46]. Chemokines are critical proinflammatory cytokines related to the host defense regulating the activation and recruitment (chemotaxis) of leukocytes or additional cell types into the neoplasia, infection, or injury sites [47]. MIP-1*β*, also known as CCL4, belongs to the chemokine family and is essential in immune responses to infection and inflammation. CCL4 is a crucial chemotactic mediator for recruiting mononuclear macrophages, natural killer cells, T lymphocytes, and cytokine production regulation [33]. Furthermore, studies have shown that CCL4 (MIP-*β*) chemokines exert vital parts within cytokine networks modulating immune and inflammatory responses of the respiratory tracts, which possibly facilitate the pathogenic mechanism of pulmonary diseases [48–50]. According to articles that assess the interstitial pulmonary disease [51], pulmonary sepsis [52], or oxidant lung damage [53] animal models, CCL4 (MIP-*β*) exerts an important part in the disease pathogenic mechanism and respiratory tract defenses. Therefore, we investigated the role of CCL4 in the sepsis-induced secondary *S. aureus* pneumonia mouse model. Firstly, we observed that in experimental sepsis-induced secondary *S. aureus* pneumonia, recombinant CCL4 could improve lung pathology and lung injury, increase the clearance rate of bacteria from the lungs and blood, and effectively promote macrophage recruitment in the lungs and reduce mortality (Figures 5(a)–5(i)). Secondly, neutrophils were the first immune cells recruited at the site of inflammation [54]. They can secrete a variety of chemokines, including IL-1*β*, IL-8, interferon-*γ* inducible protein 10(IP-10), macrophage inflammatory protein 1*α* (MIP-1*α*), and MIP-1*β*(CCL4) [55]. According to previous reports, following the release of neutrophils, MIP-1*β* (CCL4), a critical chemotactic mediator for the recruitment of monocytes/macrophages, promotes macrophages' endocytosis, leading to regression of inflammation [56]. Therefore, we further investigated whether basil polysaccharide can promote the secretion of CCL4 from neutrophils; we extracted mouse peritoneal centrioles for *in vitro* culture. The results indicated that BPS could effectively promote the secretion of CCL4 by neutrophils (Figure 5(j)). This may possibly be the molecular immune mechanism underlying the basil polysaccharide regulating the sepsis-induced secondary *S. aureus* pneumonia.

Phagocytes, especially resident macrophages and recruited neutrophils, exert an important part in immune responses at the infection sites, either in early or late stage; in addition, they express various ‘scavenger' receptors, thus clearing the senescent host cells, proteins, and foreign bacteria [57]. Our *in vitro* experiments demonstrated that pretreatment with basil polysaccharide could effectively promote the phagocytosis and killing ability of macrophages to phagocytose *S. aureus* (Figures 6(a) and 6(b)). Activating the intracellular signal transduction pathways is necessary for the interaction of host cells with foreign pathogens [58]. This study also explored the effect of BPS treatment of macrophages on changing intracellular signal transduction upon secondary infection with *S. aureus*. According to our findings, BPS remarkably promoted p38MAPK signal transduction pathway activation within macrophages after *S. aureus* challenge (Figures 6(c) and 6(d)) [34]. The abovementioned pathway participates in the ability for host cells to recognize and absorb bacteria, and the BPS-mediated enhanced abilities for macrophages to kill and swallow bacteria were partly regulated through the promoted p38MAPK signal transduction pathway activation.

Apoptosis is an essential part of normal physiological mechanisms and occurs as a homeostatic mechanism to balance cell proliferation and cell death. The initiation of apoptosis is genetically and biochemically regulated by intracellular stimuli and extracellular signals [59]. Under physiological conditions, apoptosis is necessary to eliminate pathogen-invaded cells and is involved in removing inflammatory cells; however, under pathological conditions, it is related to the development of multisystem diseases [60]. Some studies have found that the cytotoxic effect of *S. aureus* during epithelial and endothelial cell invasion is mediated through apoptosis [61, 62]. By coculturing human T lymphocytes with *S. aureus* exotoxin, Jonas et al. found that the nanomolecular concentration of toxin can cause irreversible ATP depletion of activated or resting T lymphocytes. The T lymphocyte membrane is more permeable to monovalent ions, leading to nuclear DNA degradation and cell apoptosis [63]. These studies indicate that the pathogenesis of *S. aureus* is closely associated with cell apoptosis. In this study, we found that in the sepsis-induced secondary *S. aureus* pneumonia mouse model, the lung apoptosis was significantly increased. However, treatment with basil polysaccharide can significantly reduce cell apoptosis in the lungs of mice (Figures 3(d) and 3(e)). These findings highlight another important mechanism of regulation by basil polysaccharide in sepsis-induced secondary *S. aureus* pneumonia.

The body's immune system has several functions in resisting pathogenic bacteria, regulating inflammatory response and anti-inflammatory response [64, 65]. The human immune system includes humoral immunity and cellular immunity; among cellular components, T lymphocytes represent the primary cells involved in realizing the cell-mediated immune response [66]. Studies have previously revealed that CD4 T lymphocytes are essential for the lungs to resist specific pathogens [67]. Reports also indicate that in CD4 knockout (KO) mice, the clearance rate of *S. aureus* is significantly impaired. And in *S. aureus*-mediated experimental pleurisy, CD4 T lymphocytes play an important role [68]. Therefore, we analyzed the effect of basil polysaccharide on CD4+ lymphocytes in a mouse model of sepsis-induced secondary *S. aureus* pneumonia. First, we tested the number of CD4+ lymphocytes in mouse BALF and found that basil polysaccharide can significantly increase CD4+ lymphocytes in the lungs (Figures 4(e) and 4(f)). As previous studies have also shown that BPS enhances T cell activation and antigen presentation within dendritic cells (DCs), thus enhancing the immune response and surveillance [21]. Next, we tested the CD4+ T lymphocyte subsets (Treg cells) in the BALF of experimental mice, and the results indicated that basil polysaccharide could increase the proportion of Treg cells in BALF (Figures 7(a) and 7(b)). To further illustrate that BPS affected T lymphocyte differentiation, naive CD4+ T lymphocytes were isolated from the mouse spleen for *in vitro* culture, and the result revealed that basil polysaccharide could significantly promote naive CD4+ T lymphocytes to differentiate to Treg cells (Figures 7(c) and 7(d)).

## 5. Conclusion

Collectively, in this study, we found that BPS can effectively accelerate MIP-1*β* (CCL4) secretion by neutrophils for the recruitment of monocytes/macrophages (M*Φ*) in the lung, enhance macrophage endocytosis and killing of *S. aureus* through activation of the p38MAPK signal pathway, significantly reduce cell apoptosis in the lung, and promote naive CD4+ T lymphocytes to differentiate to Treg cells. Besides, this study highlights an essential mechanism of BPS in playing a protective role in sepsis-induced secondary *S. aureus* pneumonia.

## Data Availability

The datasets used or analyzed during the current study are available from the corresponding author on reasonable request.

## Supplementary Materials

Supplementary Materials Supplementary Figure: (A) following *P. aeruginosa* infection (*n* = 15 for every group), we observed mortality rate over the 10-day period. (B, C) Lung or BALF bacterial CFU in each group after challenge with *P. aeruginosa* (*n* = 5 mice/group). (D, E) Lung injury assessment indicators such as protein in BALF, myeloperoxidase, and wet/dry weight ratio in the left lung were measured after challenge with *P*. *aeruginosa* (*n* = 5 mice/group). ^∗^*p* < 0.05, ^∗∗^*p* < 0.01, and ^∗∗∗^*p* < 0.001, upon one-way ANOVA as well as LSD multiple comparisons. Compared with *S. aureus* infection treated with basil polysaccharide group or CLP-surgery mice upon secondary *S. aureus* infection treated with basil polysaccharide group. ns: no statistical significance between the groups challenged with *P. aeruginosa*.
