# Supplementary material for: Basil Polysaccharide Reverses Development of Experimental Model of Sepsis-Induced Secondary Staphylococcus aureus Pneumonia
Source: Mediators Inflamm. 2021 May 18;2021:5596339. doi: 10.1155/2021/5596339 (PMC8149242; doi:10.1155/2021/5596339)
Supplement: Supplementary Materials — Supplementary Figure: (A) following P. aeruginosa infection (n = 15 for every group), we observed mortality rate over the 10-day period. (B, C) Lung or BALF bacterial CFU in each group after challenge with P. aeruginosa (n = 5 mice/group). (D, E) Lung injury assessment indicators such as protein in BALF, myeloperoxidase, and wet/dry weight ratio in the left lung were measured after challenge with P. aeruginosa (n = 5 mice/group). ∗p < 0.05, ∗∗p < 0.01, and ∗∗∗p < 0.001, upon one-way ANOVA as well as LSD multiple comparisons. Compared with S. aureus infection treated with basil polysaccharide group or CLP-surgery mice upon secondary S. aureus infection treated with basil polysaccharide group. ns: no statistical significance between the groups challenged with P. aeruginosa. [file 5596339.f1.pdf]

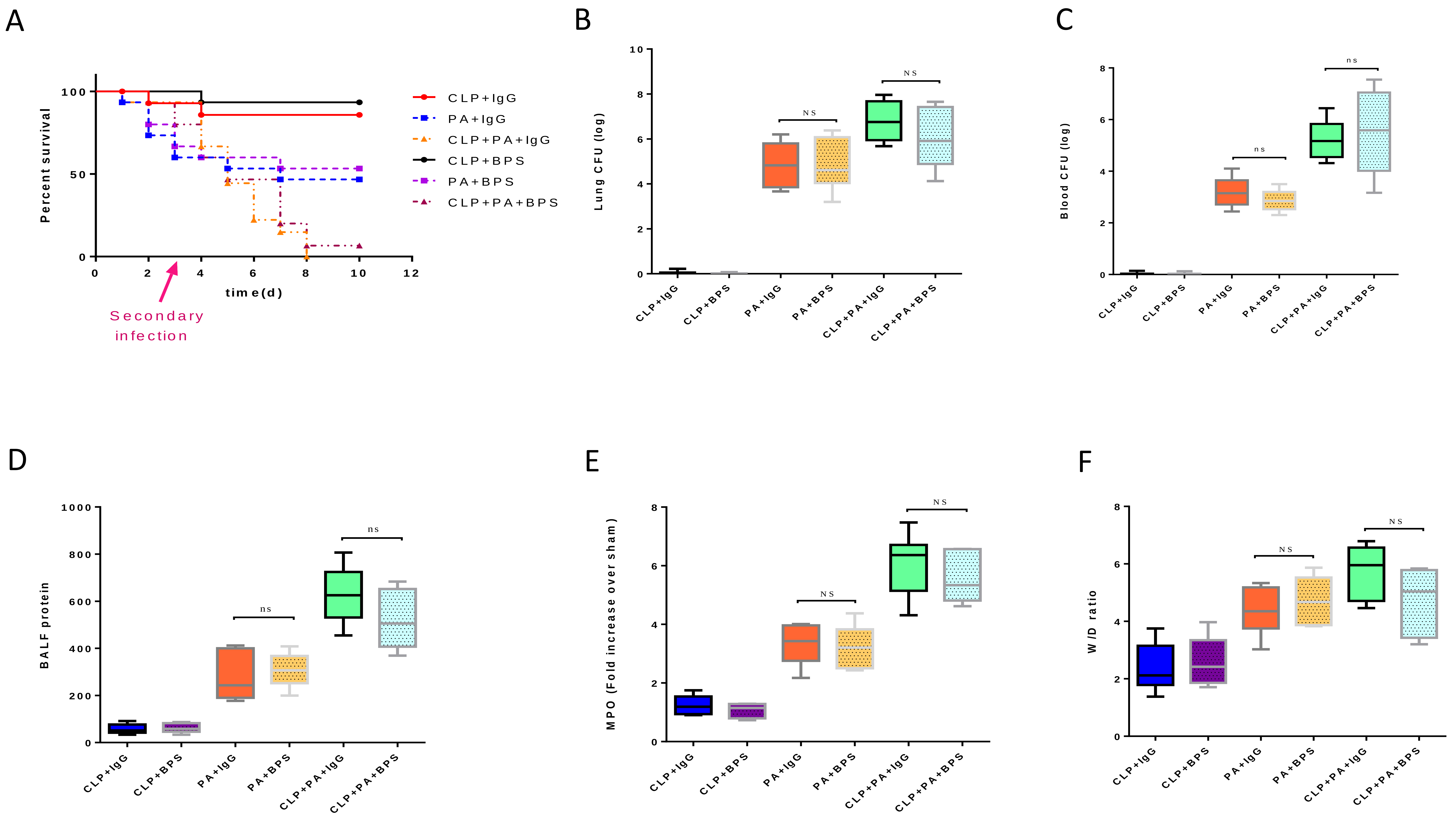

Supplementary figure

Supplementary Figure Legend

(A) Following *P. aeruginosa* infection (n = 15 for every group), we observed mortality rate over the 10-day period. (B-C) Lung or BALF bacterial CFU in each group after challenge with *P. aeruginosa* (n = 5 mice/group). (D-E) Lung injury assessment indicators such as protein in BALF, Myeloperoxidase, and Wet/dry weight ratio in the left lung were measured after challenge with *P. aeruginosa* (n = 5 mice/group).*p<0.05, **p<0.01, ***p<0.001, upon one-way ANOVA as well as LSD multiple comparisons. Compared with *S. aureus* infection treated with basil polysaccharide group or CLP-surgery mice upon secondary *S. aureus* infection treated with basil polysaccharide group. ns, no statistical significance between the groups challenged with *P. aeruginosa*.
